# Interleukin-6 Contributes to Inflammation and Remodeling in a Model of Adenosine Mediated Lung Injury

**DOI:** 10.1371/journal.pone.0022667

**Published:** 2011-07-25

**Authors:** Mesias Pedroza, Daniel J. Schneider, Harry Karmouty-Quintana, Julie Coote, Stevan Shaw, Rebecca Corrigan, Jose G. Molina, Joseph L. Alcorn, David Galas, Richard Gelinas, Michael R. Blackburn

**Affiliations:** 1 Department of Biochemistry and Molecular Biology, University of Texas Medical School at Houston, Houston, Texas, United States of America; 2 Graduate School of Biomedical Sciences, University of Texas Health Science Center at Houston, Houston, Texas, United States of America; 3 UCB Celltech, Slough, Berkshire, United Kingdom; 4 Department of Pediatrics, University of Texas Medical School at Houston, Houston, Texas, United States of America; 5 Institute for Systems Biology, Seattle, Washington, United States of America; University of Colorado Denver, United States of America

## Abstract

**Background:**

Chronic lung diseases are the third leading cause of death in the United States due in part to an incomplete understanding of pathways that govern the progressive tissue remodeling that occurs in these disorders. Adenosine is elevated in the lungs of animal models and humans with chronic lung disease where it promotes air-space destruction and fibrosis. Adenosine signaling increases the production of the pro-fibrotic cytokine interleukin-6 (IL-6). Based on these observations, we hypothesized that IL-6 signaling contributes to tissue destruction and remodeling in a model of chronic lung disease where adenosine levels are elevated.

**Methodology/Principal Findings:**

We tested this hypothesis by neutralizing or genetically removing IL-6 in adenosine deaminase (ADA)-deficient mice that develop adenosine dependent pulmonary inflammation and remodeling. Results demonstrated that both pharmacologic blockade and genetic removal of IL-6 attenuated pulmonary inflammation, remodeling and fibrosis in this model. The pursuit of mechanisms involved revealed adenosine and IL-6 dependent activation of STAT-3 in airway epithelial cells.

**Conclusions/Significance:**

These findings demonstrate that adenosine enhances IL-6 signaling pathways to promote aspects of chronic lung disease. This suggests that blocking IL-6 signaling during chronic stages of disease may provide benefit in halting remodeling processes such as fibrosis and air-space destruction.

## Introduction

Excessive remodeling and fibrosis are detrimental components of chronic lung diseases such as asthma, chronic obstructive pulmonary disease (COPD), and interstitial lung disease [1], [2], [3]. Although substantial information is available concerning the biogenesis of these disorders, the mechanisms that promote the extensive tissue remodeling seen remain enigmatic. Chronic lung diseases are largely untreatable and are the third leading cause of death in the United States [4], [5]. Thus, identifying signaling pathways involved in the regulation of progressive pulmonary remodeling may provide novel therapeutic approaches for these devastating disorders.

Extracellular adenosine is generated following cellular injury and promotes tissue protection and repair by enhancing anti-inflammatory processes and stimulating wound healing [6], [7], [8]. However, excessive adenosine production in the lung promotes tissue injury and remodeling and has been hypothesized to engage amplification pathways that contribute to disease chronicity [9]. Accordingly, adenosine levels are elevated in the lungs of humans and animal models with chronic lung disease [10], [11]. Extracellular adenosine signals through cell surface G-protein coupled adenosine receptors (A_1_R, A_2A_R, A_2B_R, and A_3_R) [12], which are also altered in the lungs of animals [11], [13] and patients [14], [15] with chronic lung disease. Recent studies suggest that the A_2B_R is responsible for regulating many of the remodeling activities of adenosine in these disorders [15], [16].

Adenosine regulates the production of the pleiotropic cytokine IL-6 in numerous cell types through engagement of the A_2B_R [15], [17]. As an inflammatory and pro-fibrotic cytokine, IL-6 is involved in the pathogenesis of lung diseases such as asthma [18], COPD [19] and idiopathic pulmonary fibrosis (IPF) [20], [21]. IL-6 signals by binding the membrane bound IL-6Rα, which then associates with the signal-transducing gp130 protein to facilitate phosphorylation of the transcription factor STAT-3 [22], [23]. Phosphorylated STAT-3 translocates to the nucleus where it regulates target gene expression. IL-6 mediated activation of STAT-3 has been implicated in several diseases [24], [25], [26]; however, little is known about the ability of adenosine to activate this pathway in the context of chronic lung disease.

The ability of adenosine to promote the production of IL-6 together with the pro-fibrotic features of this cytokine led us to hypothesize that this pathway contributes to features of chronic lung disease in environments where adenosine levels are elevated. The goal of this manuscript was to test this hypothesis using a well characterized model of adenosine-mediated lung injury, the adenosine deaminase (ADA)-deficient model [9], [16], [27], [28]. In this model, elevations in lung adenosine levels promote pulmonary inflammation, air-space destruction and fibrosis. We examined the contribution of IL-6 in this model by treating these mice with a novel IL-6 neutralizing antibody and genetically removing IL-6. Results demonstrated that IL-6 contributes to the development of pulmonary inflammation, tissue remodeling and fibrosis in ADA-deficient mice. The pursuit of mechanisms responsible for IL-6 mediated effects on fibrosis revealed an adenosine and IL-6 dependent activation of STAT-3 in airway epithelial cells. Together these findings identify a novel pathway for adenosine mediated amplification of pulmonary inflammation, remodeling and fibrosis, and highlight novel therapeutic approaches for treating chronic lung diseases.

## Methods

### Ethics Statement

Animal care was in accordance with institutional and NIH guidelines. These studies were reviewed and approved by the University of Texas Health Science Center at the Houston Animal Welfare Committee in Houston, Texas, USA. The approved animal protocol number was HSC-AWC-09-188.

### Mice

ADA-deficient mice were generated and genotyped as described previously [29]. Mice homozygous for the null *Ada* allele were designated ADA-deficient (*Ada^-/-^*), while mice heterozygous for the null *Ada* allele were designated as ADA competent mice (*Ada^+^*). All mice were on a mixed 129/C57BL/6J background, and all phenotypic comparisons were performed among littermates. To generate *Ada/IL-6* double knockout mice (*Ada/IL-6* ^-/-^), *IL-6* ^-/-^ mice congenic on a C57BL/6J background (Jackson Laboratory, Bar Harbor, ME, USA) were backcrossed with *Ada^-/-^* mice also congenic on the C57BL/6J background. Mice were housed in ventilated cages equipped with microisolator lids and maintained under strict containment protocols.

### ADA Enzyme Therapy

Polyethylene glycol modified ADA (PEG-ADA) was generated by the covalent modification of purified bovine ADA with activated polyethylene glycol as described [30]. The ADA-deficient model of lung disease was run in two manners. One to assess the impact of reversing adenosine levels, in which mice were injected with PEG-ADA on postnatal day 18, a stage when lung disease is established but reversible [27]. In this model mice were given a single injection of PEG-ADA (5 Units) and endpoints were monitored 72 hours later. However, this model cannot be used for the assessment of air-space enlargement, because of defects in alveolargenesis. Therefore, a second model of delayed adenosine elevations was used [16]. For this model, *Ada^-/-^* mice were identified at birth by screening for ADA enzymatic activity in the blood and were maintained on ADA enzyme therapy from postnatal day 1 until postnatal day 25. *Ada^-/-^* mice received intramuscular injections of PEG-ADA on postnatal days 1, 5, 9, 13, and 17 (0.625, 1.25, 2.5, 2.5, and 2.5 Units, respectively) and intraperitoneal (i.p.) injections on postnatal days 21 and 25 (5 Units each). Mice were sacrificed on day 27 after the last PEG-ADA injection (postnatal day 43). *Ada/IL-6* ^-/-^ mice were subjected to ADA enzyme therapy until postnatal day 25 and sacrificed on day 27 after treatment (postnatal day 43).

### IL-6 Neutralization

IL-6 neutralizing and isotype antibodies were provided by UCB Celltech Inc. (Slough, UK). Subcutaneous injection with IL-6 antibody (30 mg/kg) or isotype antibody (30 mg/kg) were initiated on postnatal day 26 and subsequent injections were given on day 31 and 37. Treatment groups included *Ada^-/-^* or *Ada^+^* mice receiving an IL-6 antibody, isotype antibody, PBS (vehicle), or no treatment. All mice were littermates, and both males and females were included in these experiments.

### Bronchoalveolar Lavage (BAL), Cellular Differentials, and Histology

Mice were anesthetized with avertin. The trachea was cannulated and the lungs were lavaged four times with 0.3 ml of PBS (0.95–1 ml lavage fluid recovered). Total cell counts were determined using a hemocytometer, and aliquots were cytospun onto microscope slides and stained with Diff-Quick (Dade Behring) for cellular differentials. After lavage, the lungs were infused with 10% buffered formalin at 25 cm of H_2_O pressure and fixed overnight at 4°C. Fixed lung samples were dehydrated and embedded in paraffin, and sections (5 µm) were collected on microscope slides and stained with H&E (Shandon-Lipshaw) or Masson's Trichome (EM Science) according to the manufacturer's instructions. Adenosine levels were quantified in BAL fluid using HPLC as described [31].

### Immunohistochemistry

Immunohistochemistry was performed on 5 µm sections cut from formalin-fixed, paraffin-embedded lungs. Sections were rehydrated through graded ethanols to water, endogenous peroxidases were quenched with 3% hydrogen peroxide, antigen retrieval was performed (Dako), and endogenous avidin and biotin was blocked with a Biotin-Blocking System (Dako). Slides were incubated with primary antibodies for mouse IL-6 (1∶200 dilution, 1 hr room temperature, Abcam), phospho-STAT-3 (1∶100 dilution, 4°C overnight, Abcam), or α-sma (1∶1000 dilution, 4°C overnight, mouse monoclonal, Sigma-Aldrich). All sections were incubated with ABC Elite Streptavidin reagents and appropriate secondary antibodies. Sections were developed with 3,3′-diaminobenzidine (Sigma-Aldrich) and counterstained with methyl green or hematoxylin. For α-sma staining, slides were processed with the Mouse on Mouse Kit and the Vector Red Alkaline Phosphatase Substrate Kit (Vector Laboratories).

### Western Blot Analysis

Lungs were homogenized and lysed on ice with protein lysis buffer (1 M Tris (pH7.4), 1 M NaCl, 1% Triton X-100) freshly supplemented with 1X protease inhibitor mixture (Roche Diagnostics). A 50 µg portion of total protein was electrophoresed on 10% SDS PAGE gels and transferred overnight at 4°C to Immobilon-P polyvinylidene difluoride (Millipore), and Western blotting was performed. For primary antibody detection, a rabbit polyclonal anti-mouse was used against phospho-STAT-3 (Abcam 1∶500), STAT-3 (Abcam 1∶500), and α-actin (Sigma 1∶5,000). Secondary HRP-conjugated antibodies (eBioscience) were applied for 1 hr at room temperature (for P-STAT-3, STAT-3 in 1∶1,000; and for α-actin in 1∶10,000). Signals were detected by chemiluminesce (Pierce Chemical).

### Collagen Quantification

The Sircol Collagen Assay (Biocolor Assays) was used to measure soluble collagen in BAL fluid according to the manufacturer's instructions.

### Mucus Quantification

Treatments with an IL-6 antibody (30 mg/kg) or isotype antibody (30 mg/kg) were initiated on postnatal day 18. Mice were sacrificed on day 21 and lung sections were stained with periodic acid-Schiff (PAS) as described [27]. Mucus index scores were determined using ImagePro Plus as previously described [27].

### Ashcroft Score

H&E-stained lung sections were used to determine the Ashcroft score as previously described [28], [32] on 6 mice per group using 20 fields per section in a blinded manner.

### Analysis of mRNA by Quantitative RT-PCR

Mice were anesthetized, and the lungs were rapidly removed and frozen in liquid nitrogen. Total RNA was isolated from frozen whole-lung tissue using TRIzol reagent (Invitrogen). RNA samples were then DNase treated and subjected to quantitative real-time RT-PCR. The primers, probes, and procedures for real-time RT-PCR were described previously [16], [28], [33]. Specific transcript levels for IL-6, CXCL-1, CCL2, osteopontin (OPN), IL-17, CXCL-2, TNF-α, TIMP-1, MMP-9, MMP-12, fibronectin, and α1-procollagen were determined by normalization to 18S rRNA and presented as mean normalized transcript levels using the comparative *Ct* method (2^ΔΔ*Ct*^) [34].

### Immunofluorescence

Sections were rehydrated and fixed with acetone and methanol. Endogenous fluorescence was quenched with NaBH_4_. Slides were incubated with primary antibody to fibronectin (1∶400 dilution, 1 hr room temperature, rabbit polyclonal, Sigma-Aldrich) followed by secondary antibody (1∶1000 dilution, 1 hr room temperature, donkey anti-rabbit IgG Alexa fluor 555-red, Invitrogen). Sections were covered with Vectashield anti-fade medium with DAPI (VectorLabs).

### Measurement of Vascular Permeability

Organ vascular permeability was quantified by i.p. administration of Evans blue dye (0.2 mL of 0.5% in PBS). Four hrs later, the circulation was perfused with PBS and the heart and lung were harvested. Organ Evans blue concentrations were quantified after formamide extraction (55°C overnight) by measuring absorbance at 610 nm with subtraction of reference absorbance at 450 nm. Evans blue dye contents were determined by comparison to a standard curve generated from dye dilutions.

### Determination of Alveolar Air-space Size

Alveolar air-space size was determined in pressure infused lungs by measuring mean chord lengths on H&E-stained lung sections [27]. Representative images were digitized, and a grid consisting of 53 black lines at 10.5 µm intervals was overlaid on the image. This line grid was subtracted from the lung images using Image-Pro Plus image analysis software (version 2.0; MediaCybernetics), and the resultant lines were measured and averaged to give the mean chord length of the alveolar air-spaces. The final mean chord lengths represent averages from 10 nonoverlapping images of each lung specimen. All quantitative studies were performed blinded with regard to animal genotype.

### In vitro Stimulation of Primary Alveolar Macrophages

Primary alveolar macrophages were isolated from the BAL fluid of *Ada^+^ and Ada* ^-/-^ mice. BAL fluid was centrifuged at 1,000 rpm for 10 min and cell pellets were resuspended in cell culture media (RPMI1640 containing 10% FBS and 10,000 U/ml penicillin/streptomycin). Total cells were counted using a hemocytometer and portioned into aliquots of 2.5×10^5^ cells/dish. Cells were allowed to adhere for 4 hrs at 37°C, 5% CO_2_. Cells were rinsed twice with RPMI1640 without FBS to remove non-adherent lymphocytes and neutrophils. Cells were then treated with either media alone, NECA (10 µM; Tocris Bioscience), ATP (100 µM), and deoxyATP (100 µM). For A_2B_R antagonism, cells were pre-incubated with MRS1754 (1 µM; Tocris Bioscience) for 30 min followed by either treatment with NECA or ATP. After incubation for 12 hrs at 37°C, 5% CO_2_, culture supernatants were collected and IL-6 levels were quantified using a mouse IL-6 ELISA kit (BD Biosciences).

### Statistics

Values are expressed as mean ±SEM. As appropriate, groups were compared by analysis of variance; follow-up comparisons between groups were conducted using 2-tailed Student's *t* test. A *P* value of ≤0.05 was considered to be significant.

## Results

### Elevated IL-6 and STAT-3 Activation in the Lungs of *Ada* ^-/-^ Mice

**Figure 1:**
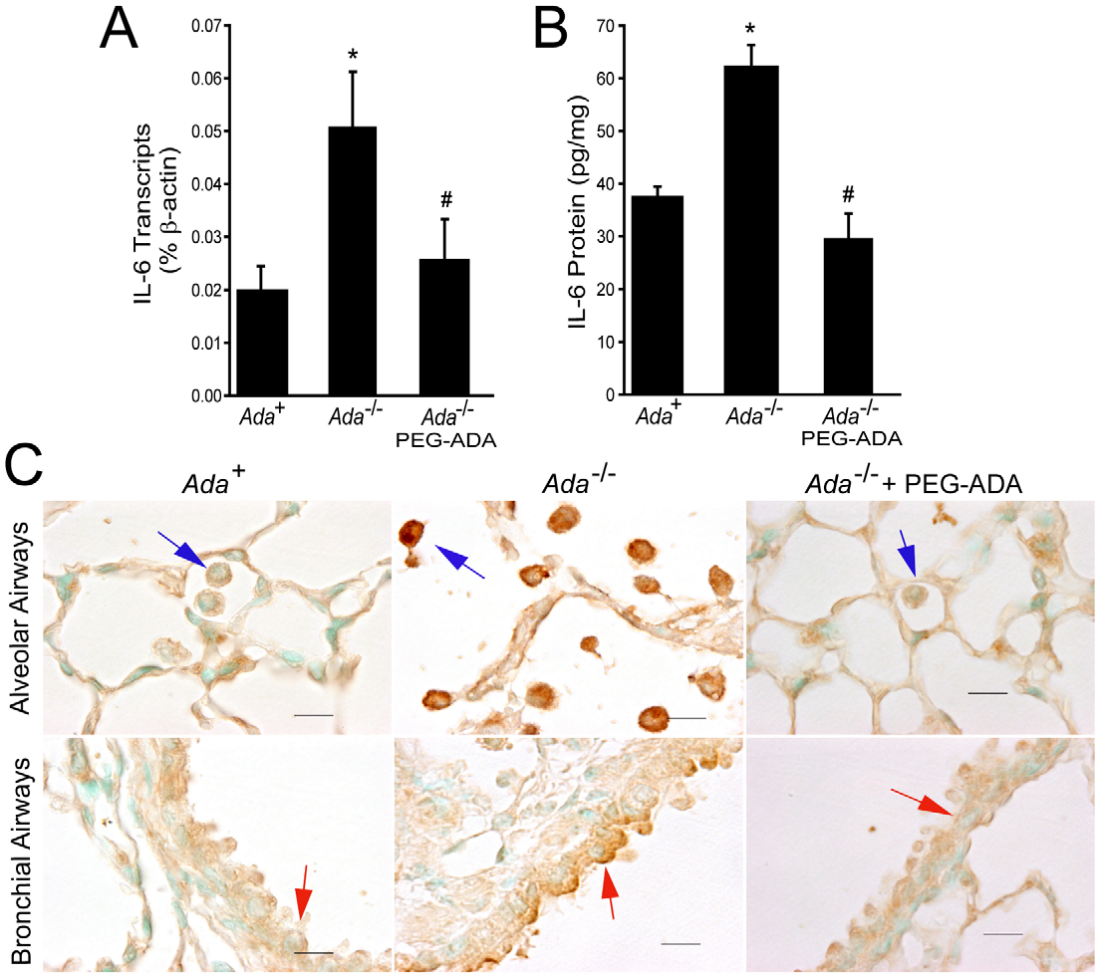
IL-6 expression in the lungs of *Ada* ^-/-^ mice and mice given ADA replacement therapy to lower adenosine levels. IL-6 expression was assessed in lung sections, BAL fluid, and whole lungs from postnatal day 18–20 *Ada* ^+^ or *Ada* ^-/-^ mice, and postnatal day 21 *Ada* ^-/-^ mice 72 hours after treatment with ADA enzyme therapy (PEG-ADA). (A) IL-6 transcript levels were measured in whole-lung RNA extracts using quantitative RT-PCR. Data are presented as the percentage of β-actin ±SEM, n≥4. (B) IL-6 protein levels were quantified in BAL fluid using ELISA. Values are presented as pg/mg of protein ±SEM, n≥4. (C) Immunohistochemical localization of IL-6 in alveolar macrophages (blue arrows) and bronchial epithelial cells (red arrows). Images are representative of 4 animals from each group. Scale bars: 10 µm. *, p≤0.05 *Ada* ^+^ vs *Ada* ^-/-^ and #, p≤0.05 *Ada* ^-/-^ vs *Ada* ^-/-^ ±PEG-ADA.

*Ada* ^-/-^ mice accumulate adenosine in their lungs in conjunction with progressive inflammation, alveolar air-space destruction and fibrosis [27], [28] making them a good model for examining adenosine-dependent features of chronic lung disease [9]. Previous studies have shown that IL-6 levels are elevated in the lungs of *Ada* ^-/-^ mice [16]; however, it is not known if these increases are dependent on adenosine elevations. To further characterize IL-6 elevations in association with adenosine elevations in this model, quantitative RT-PCR on whole-lung RNA extracts (Figure 1A) and IL-6 protein measurements in BAL fluid (Figure 1B) were determined at a stage when adenosine elevations, pulmonary inflammation, air-space destruction and fibrosis were prominent [16]. Results demonstrated increases in IL-6 transcripts and protein that were diminished following ADA enzyme replacement therapy to lower adenosine levels [28] (Figure 1A and 1B). Immunolocalization revealed alveolar macrophages and bronchial airway epithelial cells as the major sites of adenosine driven IL-6 production (Figure 1C). Consistent with this, primary macrophages isolated from the lungs of ADA-deficient mice released substantially more IL-6 in short term culture compared to macrophages isolated from the lungs of control mice (Figure S1A). Together, these studies demonstrate adenosine dependent expression of IL-6 during chronic stages of pulmonary disease in the ADA-deficient model.

Previous studies have demonstrated that engagement of the A_2B_R can promote the release of IL-6 from alveolar macrophages isolated from the lungs of patients with chronic lung disease [15]. To determine if signaling through the A_2B_R also promotes IL-6 release from alveolar macrophages from ADA-deficient mice, primary alveolar macrophages were isolated from the lavage and were stimulated with the nonselective adenosine receptor agonist NECA. NECA exposure lead to a significant increase in IL-6 release in short term culture and this increase was prevented by pretreatment with the A_2B_R antagonist MRS-1754 (Figure S1B). Interestingly, ATP also promoted the release of IL-6 from alveolar macrophages; however, this release was also blocked by pretreatment with MRS-1754 suggesting that it is the breakdown of ATP to adenosine that is mediating this response as opposed to engagement of P2 receptors. Treatment with dATP did not increase the release of IL-6. Together, these results suggest that engagement of the A_2B_R is likely responsible increasing IL-6 release from alveolar macrophages in the lungs of ADA-deficient mice.

### IL-6 Neutralization Decreases Pulmonary Inflammation in *Ada^-/-^* Mice

**Figure 2:**
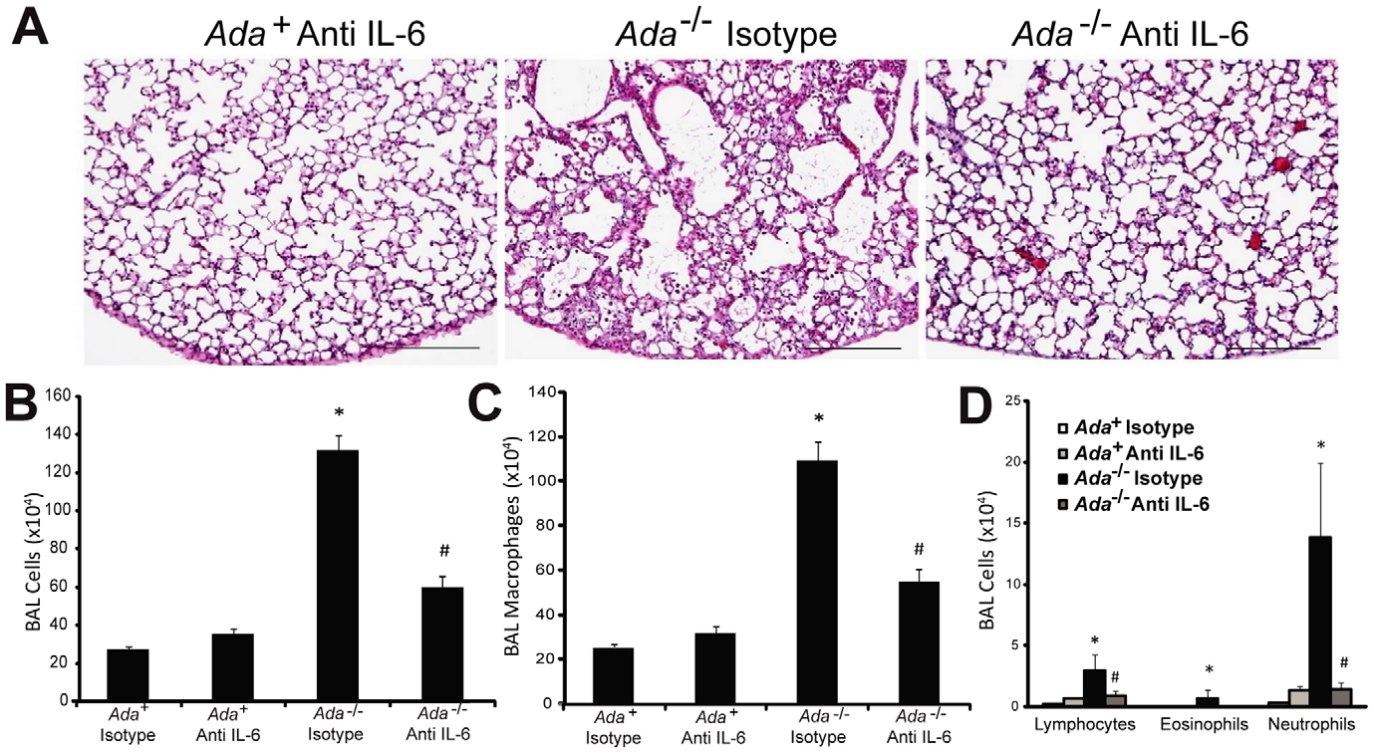
Pulmonary inflammation following the treatment of *Ada* ^-/-^ mice with IL-6 neutralizing antibodies. Mice were given subcutaneous injections of anti-IL-6 antibodies (30 mg/kg) as described in the methods. (A) Lungs from postnatal day 43 *Ada* ^+^ mice treated with IL-6 antibody (left), treatment with the isotype antibody or vehicle PBS revealed no effect, *Ada* ^-/-^ mice treated with the isotype antibody (middle) or Anti-IL-6 (right). Images are representative of 11 animals from each group. Scale bars = 200 µm. (B) Total BAL inflammatory cells numbers. Cellular differentials for (C) macrophages, (D) lymphocytes, eosinophils, and neutrophils. Data are presented as mean cell counts ±SEM, n≥11. *, p≤0.05 *Ada* ^+^ vs *Ada* ^-/-^ and #, p≤0.05 *Ada* ^-/-^ vs *Ada* ^-/-^ + Anti-IL-6.

Therapeutic approaches utilizing IL-6 neutralizing antibodies are currently in use for the treatment of inflammatory and autoimmune diseases [35]. To begin to assess the effectiveness of this approach in chronic lung disease, *Ada* ^-/-^ mice were treated with IL-6 neutralizing antibodies (Figure S2A). Exposure levels of the anti-IL-6 antibody used were consistent amongst animals and reached plasma levels of approximately 300 µg/ml (Figure S2B). Treatment with IL-6 neutralizing antibodies was associated with decreased histopathology (Figure 2A). Consistent with this, there was a significant reduction in inflammatory cells recovered from the BAL fluid on postnatal day 43 (Figure 2B) with differentials revealing reduced numbers of macrophages (Figure 2C), lymphocytes, eosinophils, and neutrophils (Figure 2D). Interestingly, treatment with IL-6 neutralizing antibodies also led to slight reductions in adenosine levels (Figure S3), which may reflect decreases in inflammation and damage in these mice. These findings demonstrate that treatment with IL-6 neutralizing antibodies can attenuate adenosine driven pulmonary inflammation.

Maintenance of pulmonary vascular barrier function plays an important role in the regulation of pulmonary inflammation [36] and the loss of pulmonary barrier function has been associated with the inflammation seen in *Ada^-/-^* mice [37]. To determine the impact of IL-6 neutralization on the loss of vascular barrier function, mice were injected with Evans blue dye 4 hrs before sacrifice on day 43 and organ levels of dye were quantified. Results demonstrated significant loss of pulmonary vascular permeability in the lungs of *Ada^-/-^* mice (Figure S4). Moreover, this loss of vascular permeability was significantly attenuated following treatment with IL-6 neutralizing antibodies. These findings suggest that IL-6 regulates the loss of pulmonary barrier function in the lungs of *Ada^-/-^* mice.

**Figure 3:**
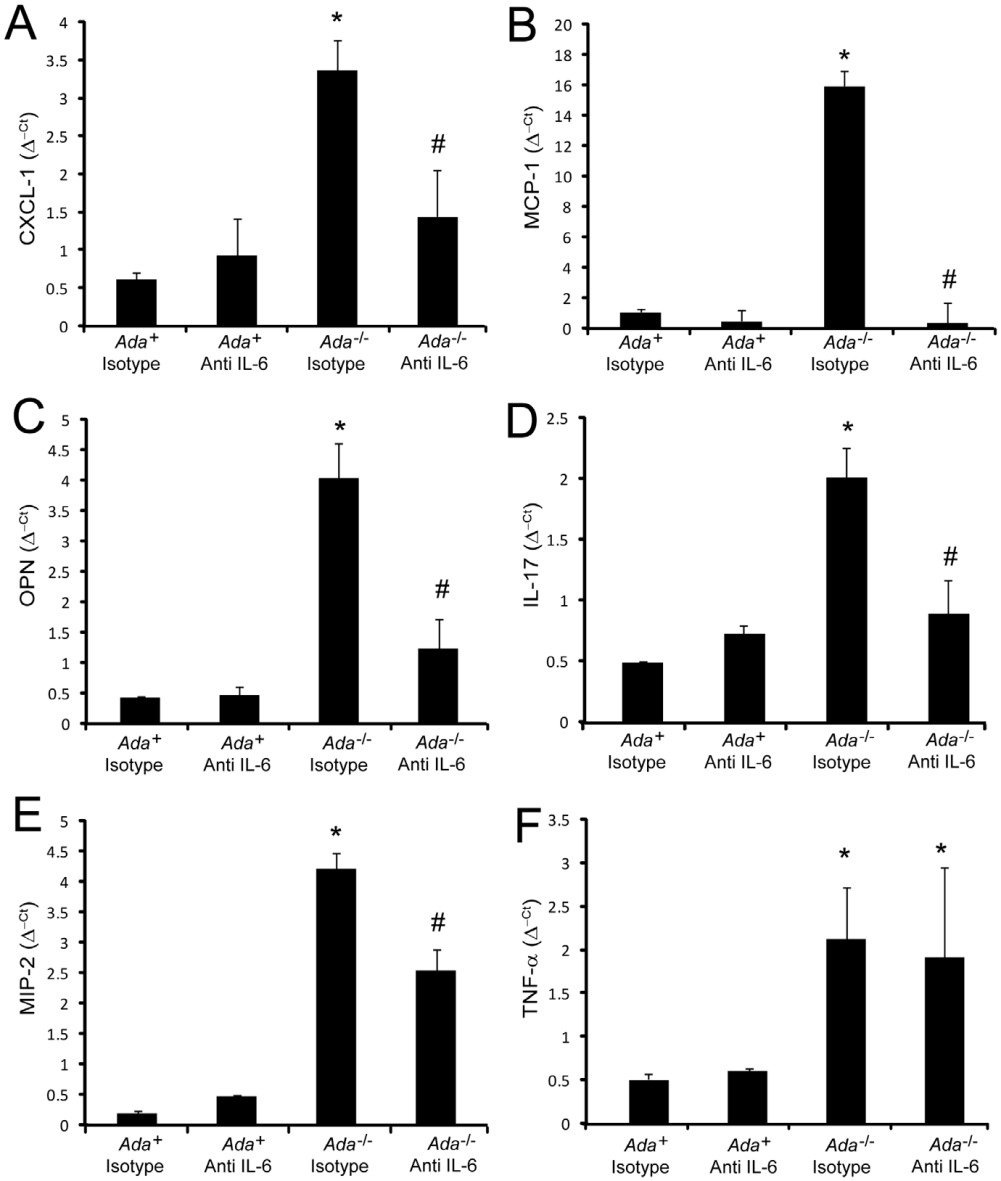
Pro-inflammatory mediators in *Ada* ^-/-^ mice treated with IL-6 neutralizing antibodies. Transcript levels of various pro-inflammatory cytokines and chemokines were measured in whole-lung RNA extracts from postnatal day 43 mice using quantitative RT-PCR. Shown are levels of (A) CXCL-1, (B) MCP-1, (C) OPN, (D) IL-17, (E) MIP-2, and (F) TNF-α. Transcripts were measured in parallel with 18S rRNA and values are presented as mean normalized transcript levels (Δ^−ct^) ±SEM, n≥4. *, p≤0.05 *Ada* ^+^ vs *Ada* ^-/-^ and #, p≤0.05 *Ada* ^-/-^ vs *Ada* ^-/-^ + Anti-IL-6.

In further pursuit of mechanisms by which IL-6 neutralization could impact inflammation in the lungs of *Ada^-/-^* mice we examined levels of key inflammatory mediators. Results revealed that the reduced pulmonary inflammation following IL-6 neutralization was associated with reductions in inflammatory mediators known to be IL-6 targets including CXCL1, MCP-1, OPN, IL-17, and MIP-2 (Figure 3A-E). In contrast, TNF-α (Figure 3F) and TGF-β (data not shown) were elevated in the lungs of *Ada* ^-/-^ mice; however, their expression was not reduced following IL-6 neutralization. These findings suggest that IL-6 influences the expression of inflammatory mediators that contribute to inflammation in the lungs of *Ada* ^-/-^ mice.

### Decreased Pulmonary Fibrosis in *Ada^-/-^* Mice Treated With IL-6 Neutralizing Antibodies

**Figure 4:**
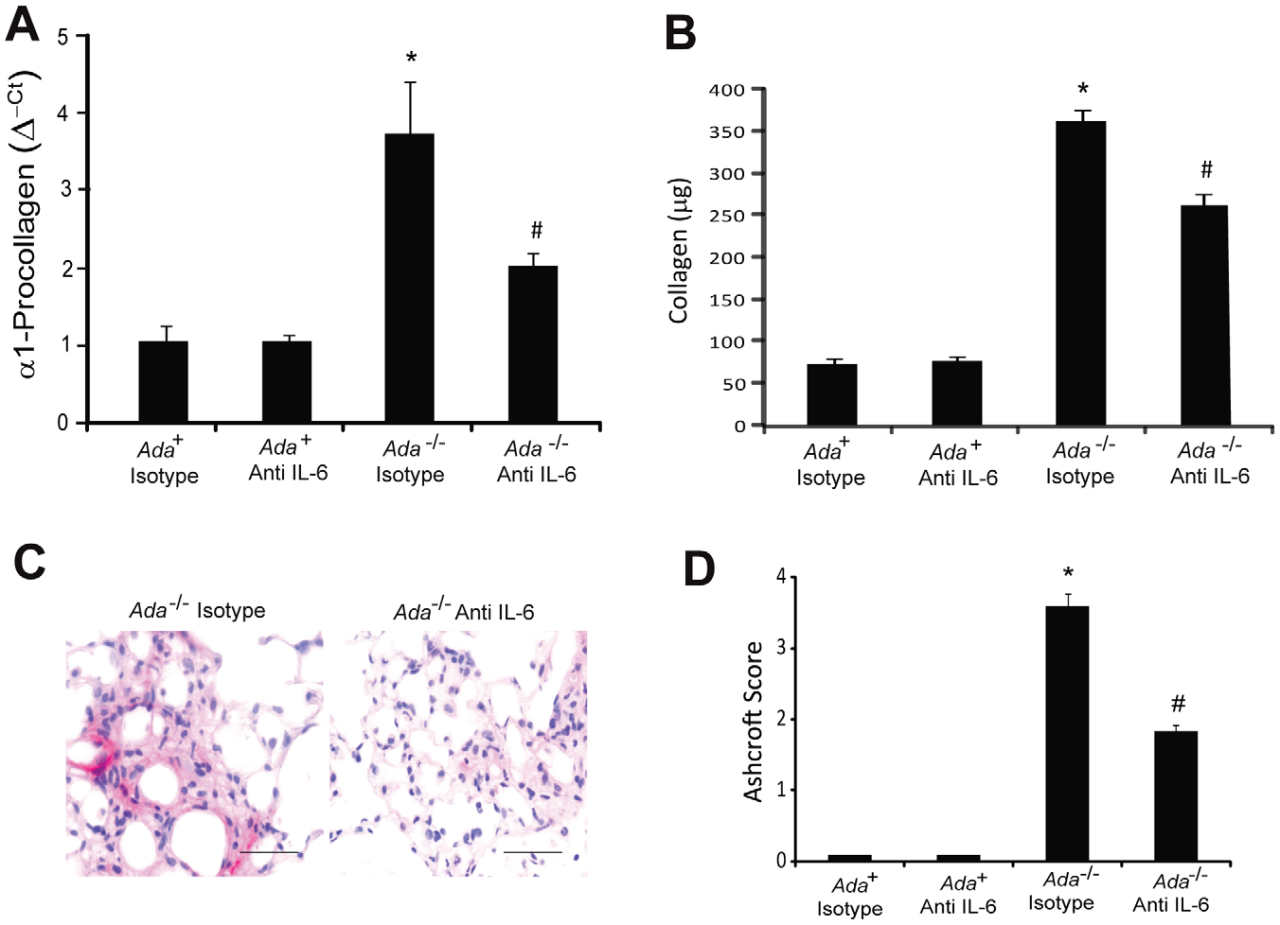
Attenuation of pulmonary fibrosis following IL-6 neutralization. (A) Decreased collagen production. Whole-lung α1-procollagen transcript levels were measured in RNA extracts from day 43 lungs using quantitative RT-PCR. Transcripts were measured in parallel with 18S rRNA and values are presented as mean normalized transcript levels (Δ^−ct^) ±SEM, n = 6. (B) Soluble collagen levels were measured using the Sircol assay and data presented as mean µg collagen/ml BAL fluid ±SEM, n≥8. (C) Lung sections were stained with an antibody against α-sma to visualize myofibroblasts (pink). Images are representative of 6 animals from each group. Scale bars: 100 µm. (D) Ashcroft scores were used as a morphometric approach to quantify overall fibrosis in the lungs, n = 6 per group. *, p≤0.05 *Ada* ^+^ vs *Ada* ^-/-^ and #, p≤0.05 *Ada* ^-/-^ vs *Ada* ^-/-^ + Anti-IL-6.

We next sought to determine if treatment with IL-6 neutralizing antibodies had an effect on the pulmonary fibrosis seen in *Ada* ^-/-^ mice [16], [28]. Treatment of *Ada* ^-/-^ mice with IL-6 neutralizing antibodies resulted in reduced lung collagen production as determined by decreased α1-procollagen transcripts (Figure 4A). Similarly, there were decreased levels of soluble collagen in the lungs of *Ada^-/-^* mice treated with IL-6 neutralizing antibodies (Figure 4B). In addition, there was decreased production and deposition of fibronectin following IL-6 neutralization (Figure S5). Moreover, myofibroblast accumulation in the distal airways was largely prevented following IL-6 neutralization (Figure 4C), and overall Ashcroft morphological fibrosis scores were improved (Figure 4D). Collectively, these findings indicate that treatment with IL-6 neutralizing antibodies attenuates pulmonary fibrosis in *Ada* ^-/-^ mice.

### IL-6 Neutralization Inhibits Alveolar Air-space Enlargement and Mucus Metaplasia in Ada^-/-^ Mice

**Figure 5:**
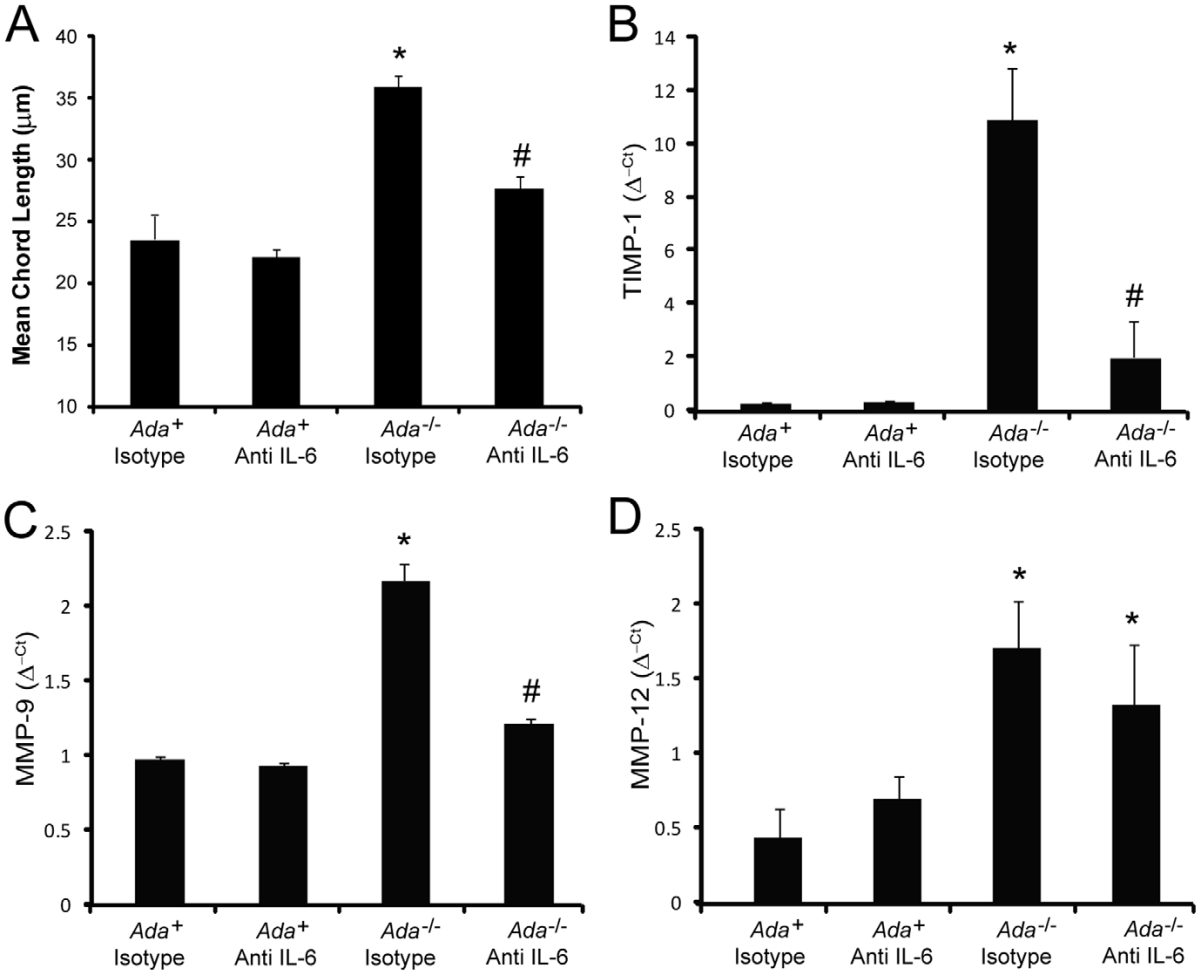
Alveolar air-space enlargement in *Ada* ^-/-^ mice following treatment with IL-6 neutralizing antibodies. Quantitative analysis of alveolar air-space size was calculated using Image-Pro analysis software on lung sections collected from mice on postnatal day 43. (A) Alveolar air-space size presented as mean chord lengths in µm ±SEM, n≥11. Alterations in mediators associated with alveolar airway space enlargement. Transcript levels of TIMP-1 (B), MMP-9 (C), and MMP-12 (E) were measured in whole-lung RNA extracts from postnatal day 43 mice using quantitative RT-PCR. Transcripts were measured in parallel with 18S rRNA and values are presented as mean normalized transcript levels (Δ^−ct^) ±SEM, n≥4. (*, p≤0.05 *Ada* ^+^ vs *Ada* ^-/-^ and #, p≤0.05 *Ada* ^-/-^ vs *Ada* ^-/-^ + Anti-IL-6.

Air-space enlargement is another adenosine driven pathological feature noted in the lungs of *Ada^-/-^* mice [27]. We next investigated the consequences of IL-6 neutralization on this feature and found that air-space enlargement was largely prevented following the treatment with IL-6 neutralizing antibodies (Figure 5A). *Ada* ^-/-^ mice exhibit increased expression of tissue inhibitor of metalloproteinase-1 (TIMP-1), matrix metalloproteinase (MMP)-9, and MMP-12 [16]. Examination of these mediators following treatment with IL-6 neutralizing antibodies revealed diminished expression of TIMP-1 and MMP-9 while MMP-12 levels remained elevated (Figure 5B-D). These results suggest that IL-6 contributes to emphysema in this model by influencing the expression of regulators of air-space destruction.

The last adenosine-driven phenotype that we examined was mucus cell metaplasia [27]. This was done by staining mucus containing cells in the bronchial airways with PAS and then using morphometry to quantify the degree of mucus in these cells. Results demonstrated that treatment with IL-6 neutralizing antibodies attenuated mucus cell metaplasia in the lungs of *Ada^-/-^* mice (Figure S6).

### *Ada/IL-6* Double Knockout Mice Exhibit Decreased Pulmonary Inflammation, Fibrosis and Air-space Destruction

**Figure 6:**
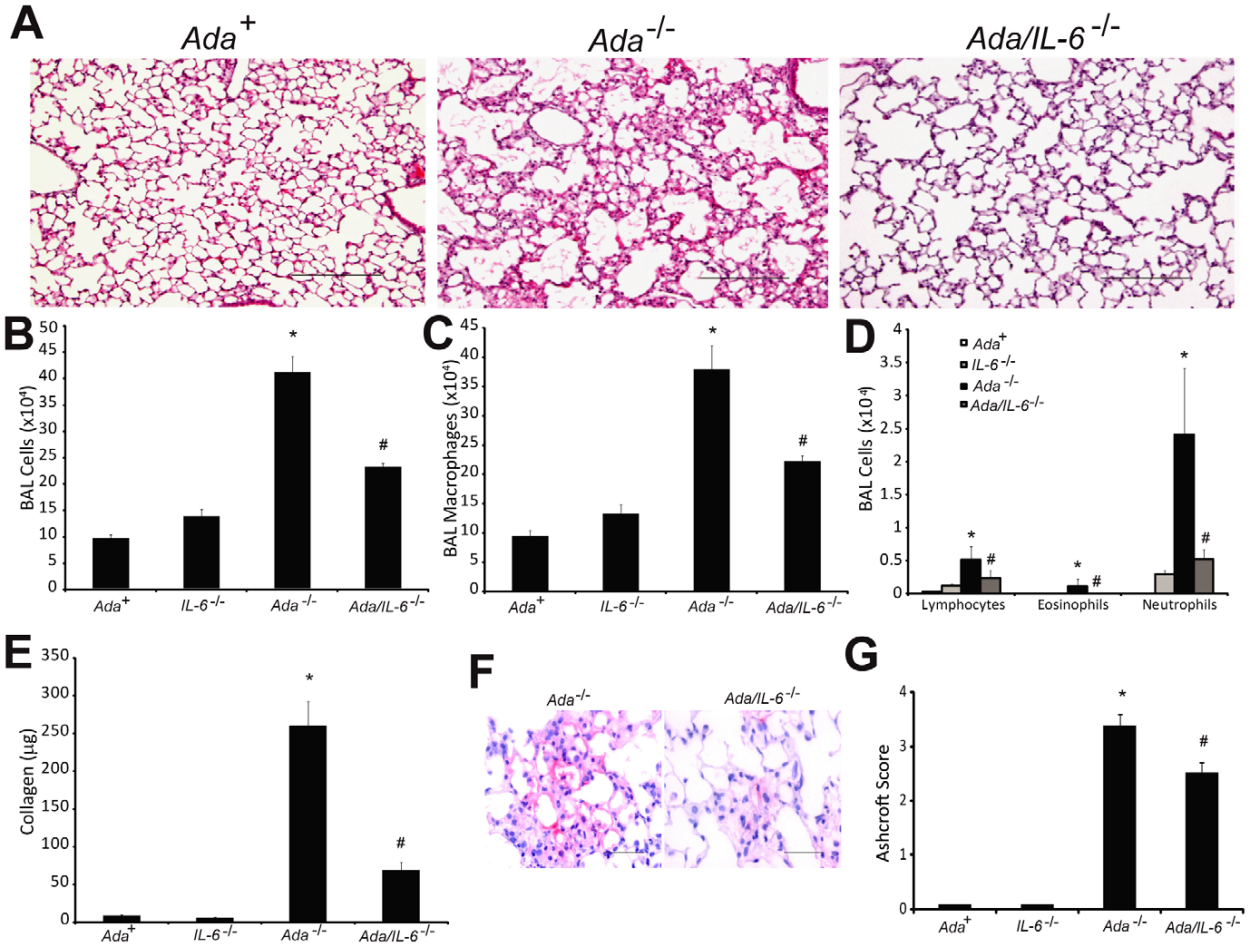
Pulmonary phenotypes following genetic removal of IL-6 in *Ada* ^-/-^ mice. (A) Lungs from postnatal day 43 *Ada* ^+^, *Ada* ^-/-^, and *Ada/IL-6* double knockout mice. Images are representative of 8 animals from each group. Scale bars: 200 µm. (B) Total cell numbers in BAL fluid were counted using a hemocytometer. BAL cells were cytospun and stained with Diff-Quick, allowing for quantification of macrophages (C) and lymphocytes, eosinophils, and neutrophils (D). Data are presented as mean cell counts ±SEM, n≥8. (E) Soluble collagen levels were measured using the Sircol Assay. Data are presented as mean µg collagen/ml BAL fluid ±SEM, n≥8. (F) Lung sections were stained with an antibody against α-sma to visualize myofibroblasts (pink). Images are representative of 6 animals from each group. Scale bars = 100 µm. (G) Ashcroft scores were used as a morphometric approach to quantify overall fibrosis in the lungs, n = 6. *, p≤0.05 *Ada* ^+^ vs *Ada* ^-/-^ and #, p≤0.05 *Ada* ^-/-^ vs. *Ada/IL-6* ^-/-^.

**Figure 7:**
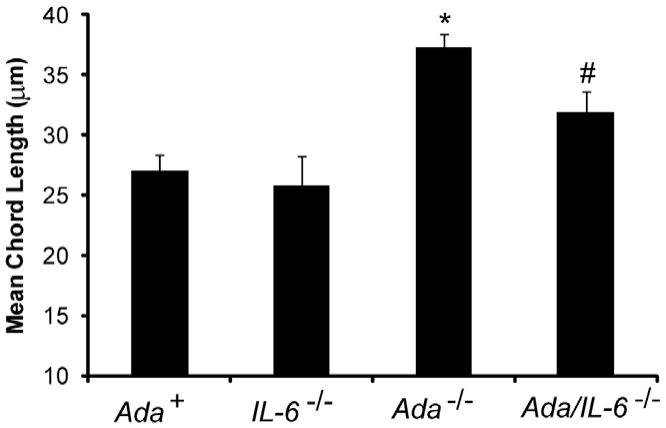
Alveolar air-space size in *Ada/IL-6* double knockout mice. Quantitative analysis of alveolar air-space size was calculated using Image-Pro analysis software on lung sections collected from mice on postnatal day 43. Data are presented as mean chord lengths in µm ±SEM, n≥8. *, p≤0.05 *Ada* ^+^ vs *Ada* ^-/-^ and #, p≤0.05 *Ada* ^-/-^ vs. *Ada/IL-6* ^-/-^.

Our results suggest that treatment of *Ada^-/-^* mice with IL-6 neutralizing antibodies leads to attenuation of many of the pulmonary phenotypes seen in this model. To gain addition insight into the role of IL-6 in this model we generated *Ada/IL-6* double knockout mice and examined key pulmonary phenotypes [16], [27]. Lung histopathology demonstrated reduced pulmonary injury in *Ada/IL-6* double-knockout mice (Figure 6A) together with a reduction in pulmonary inflammation, including decreased levels of macrophages, lymphocytes, eosinophils and neutrophils (Figure 6B-D). Examination of metrics of pulmonary fibrosis revealed reduced collagen production (Figure 6E), diminished myofibroblast accumulation (Figure 6F), and reduced Ashcroft fibrotic scores (Figure 6G). In addition, alveolar air-space enlargement, was significantly diminished by the genetic removal of IL-6 (Figure 7). Collectively, these results demonstrate that genetic removal of IL-6 in *Ada^-/-^* mice leads to attenuated pulmonary inflammation, fibrosis and air-space destruction, further implicating IL-6 signaling in mediating aspects of adenosine driven chronic lung disease.

### Adenosine and IL-6 Mediate STAT-3 Activation in Airway Epithelial Cells

**Figure 8:**
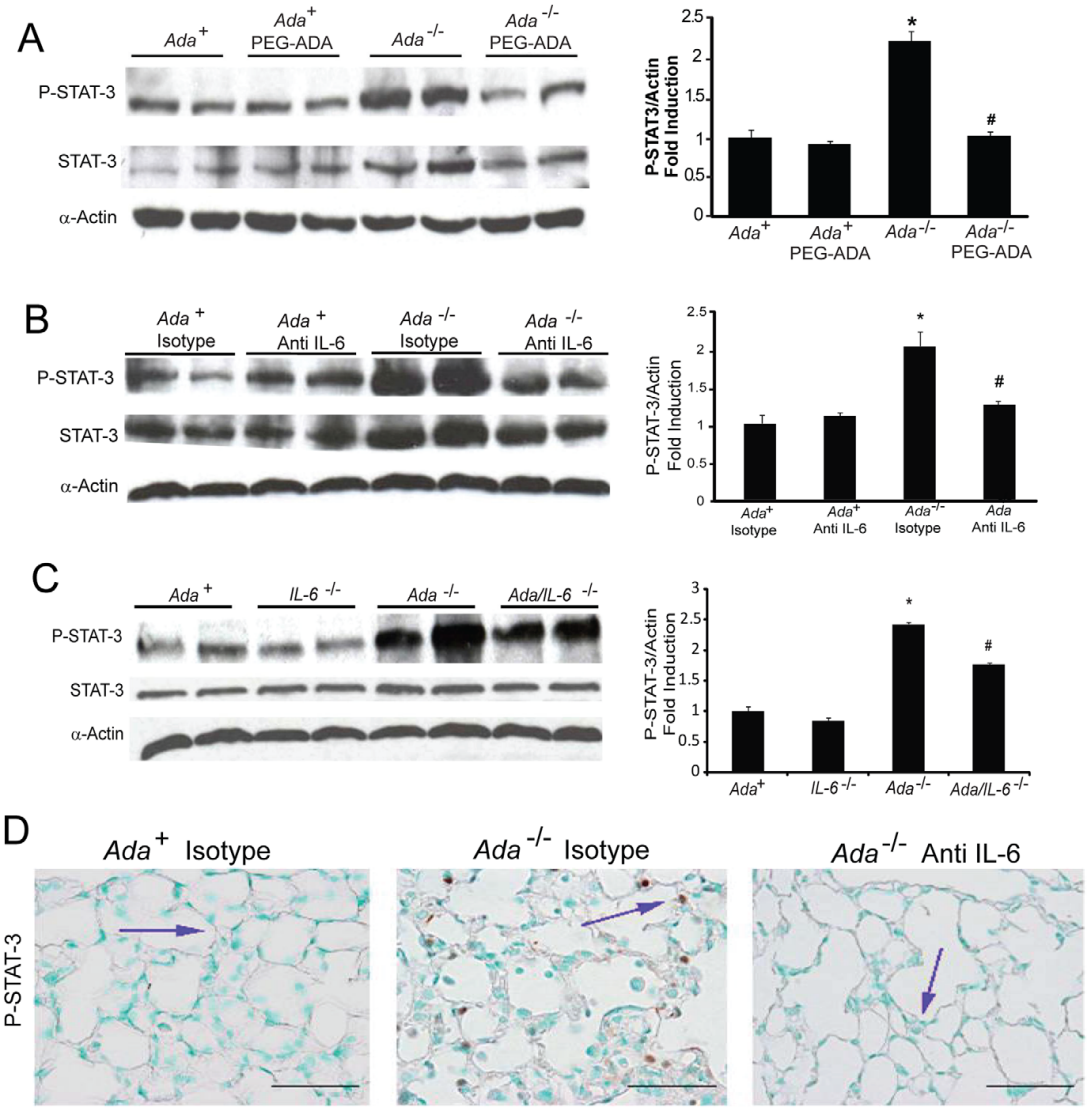
STAT-3 activation in the lungs of *Ada^-/-^* mice. STAT-3 activation was measured in whole-lung extracts using western blot analysis with a phospho-STAT-3 antibody. Total STAT-3 levels were also examined and α-actin levels were used as a loading control. Duplicate mice from each condition are shown. Phospho-STAT-3 band intensity was quantified using Image J analysis and values are presented as the percentage of α-actin ±SEM, n≥4. *, p≤0.05 *Ada* ^+^ vs *Ada* ^-/-^ and #, p≤0.05 *Ada* ^-/-^ vs. treatment or genetic alteration. Conditions examined include (A) *Ada^+^* and *Ada^-/-^* mice treated with ADA enzyme therapy (PEG-ADA), (B) day 43 *Ada^+^* and *Ada^-/-^* mice treated with IL-6 neutralizing antibodies, and (C) *Ada^+^*, *Ada^-/-^*, *IL-6^-/-^* and *Ada/IL-6^-/-^* mice. (D) Immunohistochemical localization of P-STAT-3 in alveolar epithelial cells (blue arrows) of *Ada^-/-^* mice. P-STAT-3 was not detected in lung sections from *Ada^+^* mice and *Ada^-/-^* mice treated with Anti-IL-6. Images are representative of 4 animals from each group. Scale bars = 100 µm.

IL-6 activates transcription in part through a pathway that leads to the phosphorylation of the transcription factor STAT-3, which when activated translocates to the nucleus to activate target genes [23]. To determine if STAT-3 was activated in the lungs of *Ada^-/-^* mice in an adenosine driven manner we monitored the levels of phosphorylated STAT-3 (P-STAT-3) using Western blot analysis. Our results demonstrated increased P-STAT-3 in lung homogenates from *Ada* ^-/-^ mice, which were diminished following PEG-ADA treatment to lower adenosine levels (Figure 8A). These findings suggest that STAT-3 is activated in an adenosine driven manner in the lungs of *Ada^-/-^* mice. We next examined the contribution of IL-6 to the activation of STAT-3 in this model by monitoring P-STAT-3 in the lungs of mice treated with IL-6 neutralizing antibodies or following genetic removal of IL-6. Results demonstrated a substantial reduction of STAT-3 activation in the lungs of *Ada^-/-^* mice treated with IL-6 neutralizing antibodies (Figure 8B) or following the genetic removal of IL-6 (Figure 8C). These results indicate that IL-6 is the dominant activator of STAT-3 in the lungs of *Ada* ^-/-^ mice.

Next, we performed immunolocalization experiments in lung sections to localize STAT-3 activation. We found that alveolar airway epithelial cells were the major cell type exhibiting nuclear P-STAT-3 in the lungs of *Ada^-/-^* mice (Figure 8D). Moreover, evidence of STAT-3 activation in alveolar airway epithelial cells was dramatically attenuated in *Ada^-/-^* mice treated with IL-6 neutralizing antibodies. Similar results were noted in the lungs of *Ada^-/-^* mice treated with ADA enzyme therapy to lower adenosine levels or in *Ada/IL-6* double knockout mice (data not shown). These findings suggest that alveolar airway epithelial cells are a major target of adenosine and IL-6 mediated STAT-3 activation in the lungs of *Ada^-/-^* mice.

## Discussion

Generation of adenosine following cellular damage is an important component of the injury response where it plays active roles in processes that protect cells and tissues, limit inflammation and promote wound healing [6], [8], [9]. However, excessive adenosine production and signaling have been implicated in the amplification of remodeling responses that occur in chronic lung diseases such as COPD and IPF [9]. The mechanisms by which adenosine promotes chronic tissue remodeling are not well understood. In the current study, we show that the pro-fibrotic mediator IL-6 is produced by alveolar macrophages in the airways of ADA-deficient mice that develop features of chronic lung disease in response to elevations in adenosine [27]. Furthermore, we demonstrate that pharmacologic inhibition or genetic removal of IL-6 attenuates pulmonary inflammation, fibrosis and air-space destruction in this model of adenosine driven lung disease. In addition, STAT-3 was found to be activated in the lungs of ADA-deficient mice in conjunction with IL-6 elevations, and blocking IL-6 signaling led to diminished STAT-3 activation, implicating this pathway in the regulation of IL-6 induced pathologies in this model. These findings identify a novel mechanism by which adenosine can promote amplification pathways that lead to disease progression. Furthermore, these findings suggest that IL-6 neutralizing antibodies might be useful in the treatment of chronic lung diseases such as COPD and IPF.

IL-6 production in the lungs of ADA-deficient mice was found to be abundant in alveolar macrophages and we demonstrated that antagonism of the A_2B_R could block IL-6 release from these cells in vitro. These findings are consistent with observations that engagement of the A_2B_R can promote IL-6 production and release from alveolar macrophages isolated from the lungs of patients with COPD and IPF [15], and human lung fibroblasts [17]. Furthermore, these findings are consistent with recent experiments from our lab demonstrating reduced IL-6 production in alveolar macrophages in the lungs of A_2B_R knockout mice exposed to bleomycin [37]. Collectively, these findings support the model that elevations in adenosine promote IL-6 production from alveolar macrophages through engagement of the A_2B_R in chronic lung disease. However, it is important to note that different observations have been made regarding A_2B_R mediated IL-6 production in acute lung injury models [38], [39], [40]. Eckel and colleagues demonstrated that genetic removal of the A_2B_R was associated with increased IL-6 production in models of acute lung injury [39]. These discrepancies may be attributed to secondary effects associated with enhanced inflammation in acute lung injury models in which the A_2B_R has been completely deleted or to differences in cell types that are present in acute or chronic lung disease models. Understanding the cellular source and mechanism of IL-6 production in specific lung disorders will be essential to deciphering the effects of A_2B_R based therapies.

A major finding of this study was that treatment with IL-6 neutralizing antibodies or the genetic removal of IL-6 could decrease inflammation in the lungs of ADA-deficient mice. This occurred in conjunction with reduced expression of inflammatory mediators such as CXCL-1, MCP-1, MIP-2, OPN and IL-17. We chose to examine these particular mediators because of published evidence that IL-6 can regulate them in models of lung injury [21], [41], [42], [43], [44]. Thus, reductions in these immunomodulatory molecules could provide mechanistic insight into the diminished inflammation seen following IL-6 blockade, placing IL-6 early in a cascade of events contributing to adenosine-induced lung inflammation. However, it is important to note that not all of the mediators examined were completely reduced following IL-6 blockade suggesting their regulation may be more complex. In addition to the reduction in mediator production, the loss of barrier function in this model, which was also regulated by IL-6, could contribute to the effects seen on inflammation. It is difficult to decipher the contribution of these inflammatory responses to the remodeling responses seen; however, macrophages and neutrophils are known to produce mediators that contribute to both fibrosis [1] and air-space destruction [45], [46]. Thus, although cause and effect cannot be established, our results suggest that blocking IL-6 signaling during chronic stages of lung disease can attenuate the production of key effector molecules and the loss of barrier function, both of which likely impact the remodeling processes seen.

Results from our study also demonstrate that IL-6 contributes to pulmonary fibrosis in ADA-deficient mice. These are the first studies to demonstrate the ability to halt the progression of fibrosis using IL-6 neutralizing antibodies suggesting IL-6 as a potential therapeutic target for treating pulmonary disorders where fibrosis is abundant. In further support of this, IL-6 levels have been shown to be elevated in various pulmonary disorders associated with fibrosis, including IPF [47], asthma [48] and COPD [49]. Moreover, *in vitro* studies have demonstrated pro-fibrotic activities of IL-6 on fibroblasts and myofibroblasts [17], [50], [51], which are considered major effector cells in pulmonary fibrosis [1]. In addition, IL-6 deficiency attenuates fibrosis following intra-tracheal bleomycin exposure [21]. Collectively, these findings support a pro-fibrotic role for IL-6 in chronic lung disease.

In addition to reduced fibrosis, we found that treatment with IL-6 neutralizing antibodies or genetic removal of IL-6 decreased alveolar air-space destruction in ADA-deficient mice. Alveolar air-space destruction is a feature seen in COPD patients exhibiting emphysema [45]. Moreover, IL-6 levels are elevated in patients with COPD [49], where it is thought to influence inflammatory responses that contribute to pulmonary hypertension associated with this disorder [52]. Our results suggest that IL-6 may also play an active role in the regulation of air-space destruction. This could be due to the regulation of inflammation, or by influencing levels of proteases or anti-proteases involved in the regulation of air-space integrity. For example, IL-6 has been shown to regulate the expression of various MMPs [53] and inhibitors of MMPs such as TIMP-1 [54]. These mediators are implicated in emphysema [45], [46], and we demonstrate that treatment with IL-6 neutralizing antibodies is associated with decreased expression of MMPs and TIMP-1. Additional studies are needed to identify the specific pathways by which IL-6 influences air-space destruction.

In pursuit of cells that are activated by IL-6, we probed lung sections from ADA-deficient mice for nuclear phospho-STAT-3 immunolocalization. Surprisingly, we found that the most abundant STAT-3 activation was found in alveolar airway epithelial cells. Moreover, this activation was lost upon treatment with ADA enzyme therapy to lower lung adenosine levels as well as by genetic or pharmacologic blockade of IL-6, suggesting that adenosine mediated IL-6 production is the major pathway responsible for STAT-3 activation in this model. Injury of airway epithelial cells is thought to play a major role in the pathogenesis of both COPD and IPF [1], [45]. Apoptosis of airway epithelial cells contributes to the loss of air-space integrity in COPD [45] and persistent injury of these cells is thought to contribute to the chronic remodeling and fibrogenesis seen in IPF [1]. Thus, activation of STAT-3 in airway epithelial cells may regulate processes that contribute to distal airway remodeling in these chronic lung diseases. The consequences of adenosine and IL-6 driven STAT-3 activation in airway epithelial cells are currently under investigation; however, our findings identify this cell type as an important target of this pathway during chronic stages of disease where adenosine is elevated. Previous studies have demonstrated activation of STAT-3 in airway epithelial cells where it was found to play a protective role in acute lung injury processes by regulating surfactant production [25], [55], [56], [57]. Thus, the role of IL-6 signaling may differ between acute and chronic stages of lung disease. This concept has been observed in other disorders including the progression of colon cancer, where it has been proposed that classical IL-6 signaling predominates in acute stages of disease, while IL-6 *trans* signaling becomes important in chronic stages [58]. Understanding the mechanisms and timing behind the protective and remodeling activities of IL-6 in various lung disorders will be important if IL-6 blocking strategies are to be pursued for the treatment of these disorders.

In conclusion, our studies demonstrate that IL-6 is an important mediator of inflammation and remodeling in a chronic lung disease model driven by adenosine [9], [15]. Adenosine and IL-6 are also elevated in models and humans with chronic lung disease [9], [10], [11], [59] and we speculate that adenosine elevations in chronic environments serve to elevate IL-6 levels that in turn activate inflammatory cascades and promote remodeling processes such as fibrosis and air-space destruction. Blocking IL-6 signaling during chronic stages of disease may thus provide benefit in halting remodeling processes such as fibrosis and air-space destruction.

## Supporting Information

Figure S1 **Enhanced IL-6 release from alveolar macrophages is mediated by the A_2B_R.** Primary alveolar macrophages were isolated from the lungs of ADA-competent (*Ada^+^*) or ADA-deficient mice (*Ada^-/-^*) on postnatal day 18 and placed in short term culture. (A) IL-6 levels measured in culture media after 12 hrs. Data are presented as mean pg/ml±SEM. n = 5. *, p≤0.05 *Ada* ^+^ vs *Ada* ^-/-^ . (B) Cells were stimulated with NECA (10 µM), ATP (100 µM) or dATP (100 µM) for 12 hrs. For A_2B_R antagonism, cells were pretreated for 15 min with MRS-1754 (1 µM) before the addition of agonist. IL-6 levels in media were quantified using ELISA and data are presented as mean fold increases ±SEM, n = 5. * p≤0.01 compared to media alone; # p≤0.01 compared to NECA; # # p≤0.05 compared to ATP.
(TIF)

Figure S2 **Treatment with IL-6 neutralizing antibodies.** (A) Schematic diagram illustrating experimental design. *Ada^+^* and *Ada* ^-/-^ mice were identified at birth and placed on ADA enzyme replacement therapy (PEG-ADA) until postnatal day 25. Mice were treated subcutaneously with an IL-6 neutralizing antibody at postnatal day 26, 31, and 37. Group controls included: no treatment and isotype antibody. (B) Pharmacokinetic analysis revealing IL-6 antibody exposure levels in the plasma. Data are presented as mean µg/ml IL-6 antibody±SEM, n≥11.
(TIF)

Figure S3 **Lavage adenosine levels.** Adenosine levels were quantified in 100 µl aliquots of BAL fluid using HPLC. Values are presented as mean adenosine concentrations (nM) ±SEM. *, p≤0.05 compared to *Ada* ^+^.
(TIF)

Figure S4 **Vascular barrier function following treatment with IL-6 neutralizing antibodies.** *Ada^+^* and *Ada* ^-/-^ mice were treated subcutaneously with IL-6 neutralizing antibodies as described in the methods. On day 43, mice were injected with Evans blue dye. 4 hrs later, mice were anesthetized, perfused and lungs and hearts were removed. (A) Whole mounts demonstrating increased Evans blue tissue uptake in the lungs of *Ada^-/-^* mice and reduced uptake in *Ada^-/-^* treated with IL-6 neutralizing antibodies (Anti IL-6). Images are representative of 4 mice. (B) Organs were extracted in formamide and dye concentrations were determined in lungs and hearts. Data are presented as mean µg/lung ±SEM. n = 4. *, p≤0.05 *Ada* ^+^ vs *Ada* ^-/-^ and #, p≤0.05 *Ada* ^-/-^ vs *Ada* ^-/-^ + Anti-IL-6.
(TIF)

Figure S5 **Decreased fibronectin in the lungs of** ***Ada*** **^-/-^ mice treated with IL-6 neutralizing antibodies.** Analyses were on postnatal day 43. (A) Whole-lung fibronectin transcript levels were measured using quantitative RT-PCR. Data are presented as mean normalized 18S rRNA transcript levels (Δ^−ct^) ±SEM, n≥4. *, p≤0.05 *Ada* ^+^ vs *Ada* ^-/-^ and #, p≤0.05 *Ada* ^-/-^ vs *Ada* ^-/-^ + Anti-IL-6. (B) Decreased fibronectin deposition visualized by fibronectin immunofluorescence (red) blue represents dapi stained nuclei. Images are representative of 8 animals from each group. Scale bars: 200 µm.
(TIF)

Figure S6 **Decreased mucus cell metaplasia following treatment with IL-6 neutralizing antibodies.** *Ada^+^* and *Ada* ^-/-^ mice were treated subcutaneously with an IL-6 neutralizing antibodies as described in the methods. Lung sections from day 43 mice were subjected to periodic acid-Schiff (PAS) staining that stained mucus pink. (A) Representative views of bronchial airways from 6 mice in each group. (B) Image Pro software was used to quantify the degree of periodic acid-Schiff staining in bronchial epithelial cells and data are presented as a mean Mucus Index ±SEM. n = 6; p≤0.05 *Ada* ^+^ vs *Ada* ^-/-^ and #, p≤0.05 *Ada* ^-/-^ vs *Ada* ^-/-^ + Anti-IL-6.
(TIF)
