# Supplementary figures and images for: Interleukin-6 Contributes to Inflammation and Remodeling in a Model of Adenosine Mediated Lung Injury
Source: PLoS One. 2011 Jul 25;6(7):e22667. doi: 10.1371/journal.pone.0022667 (PMC3143181; doi:10.1371/journal.pone.0022667)

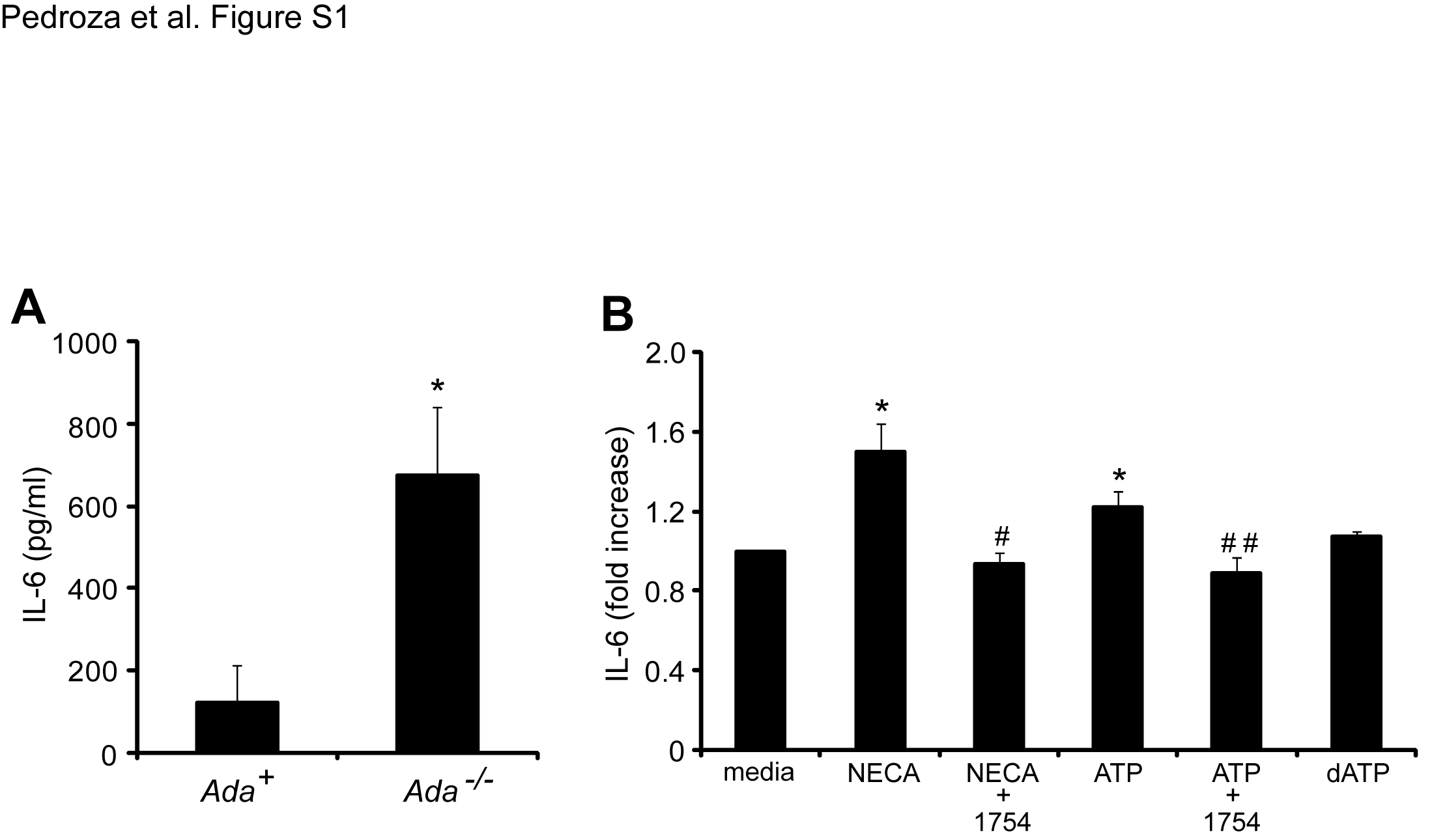

Supplement: Figure S1 — Enhanced IL-6 release from alveolar macrophages is mediated by the A2BR. Primary alveolar macrophages were isolated from the lungs of ADA-competent (Ada+) or ADA-deficient mice (Ada-/-) on postnatal day 18 and placed in short term culture. (A) IL-6 levels measured in culture media after 12 hrs. Data are presented as mean pg/ml±SEM. n = 5. *, p≤0.05 Ada + vs Ada -/- . (B) Cells were stimulated with NECA (10 µM), ATP (100 µM) or dATP (100 µM) for 12 hrs. For A2BR antagonism, cells were pretreated for 15 min with MRS-1754 (1 µM) before the addition of agonist. IL-6 levels in media were quantified using ELISA and data are presented as mean fold increases ±SEM, n = 5. * p≤0.01 compared to media alone; # p≤0.01 compared to NECA; # # p≤0.05 compared to ATP. (TIF) [file pone.0022667.s001.tif]

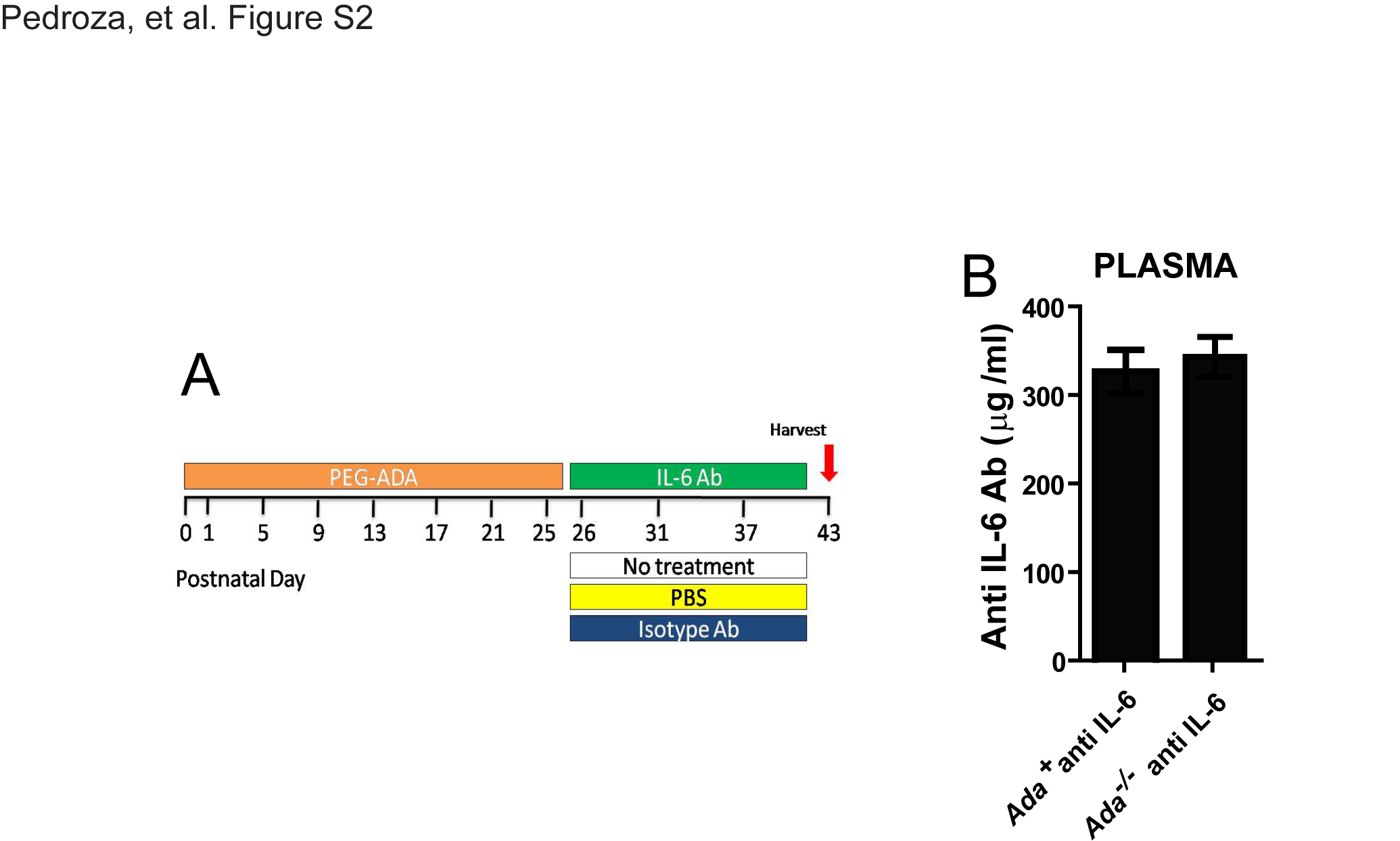

Supplement: Figure S2 — Treatment with IL-6 neutralizing antibodies. (A) Schematic diagram illustrating experimental design. Ada+ and Ada -/- mice were identified at birth and placed on ADA enzyme replacement therapy (PEG-ADA) until postnatal day 25. Mice were treated subcutaneously with an IL-6 neutralizing antibody at postnatal day 26, 31, and 37. Group controls included: no treatment and isotype antibody. (B) Pharmacokinetic analysis revealing IL-6 antibody exposure levels in the plasma. Data are presented as mean µg/ml IL-6 antibody±SEM, n≥11. (TIF) [file pone.0022667.s002.tif]

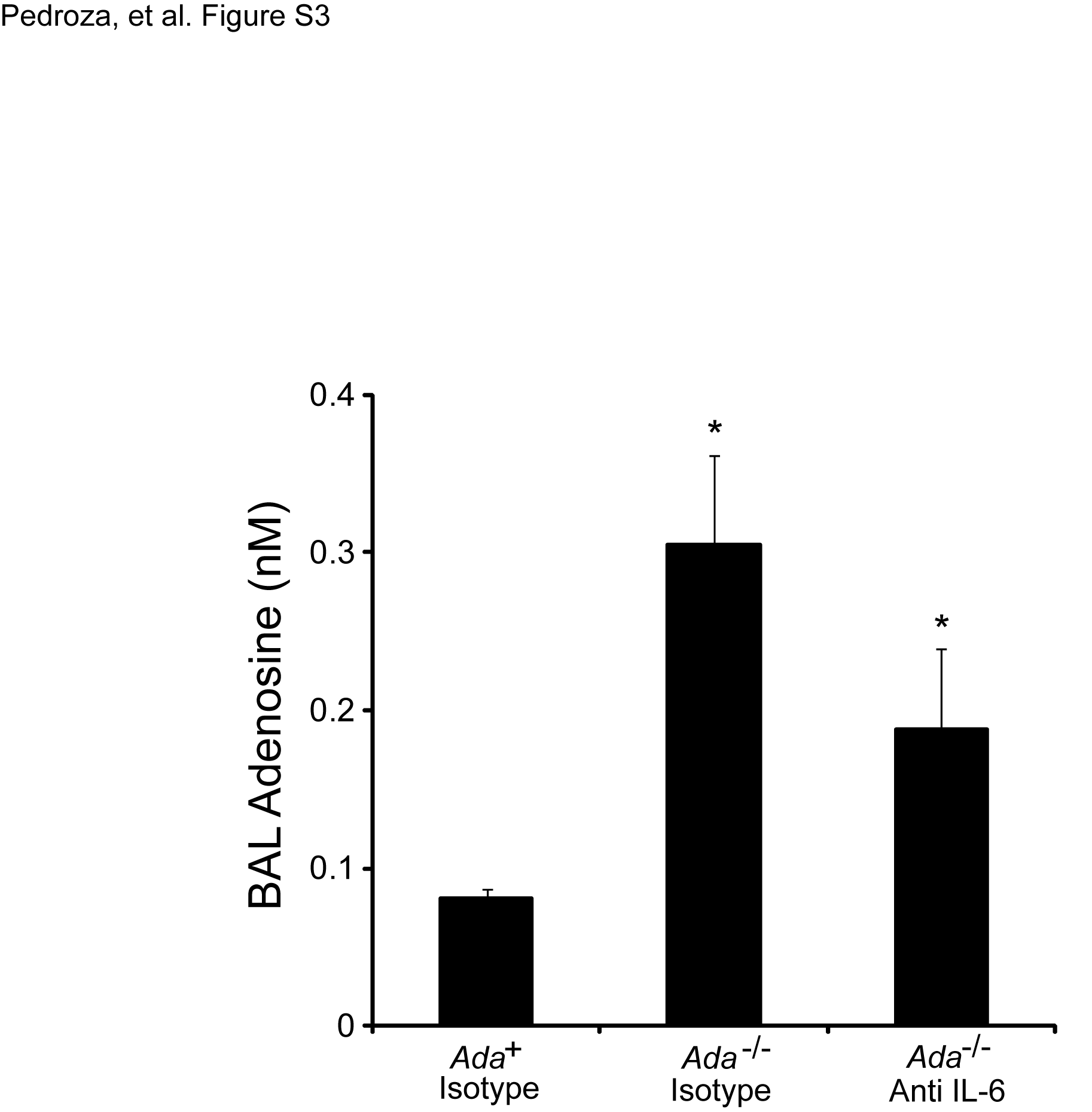

Supplement: Figure S3 — Lavage adenosine levels. Adenosine levels were quantified in 100 µl aliquots of BAL fluid using HPLC. Values are presented as mean adenosine concentrations (nM) ±SEM. *, p≤0.05 compared to Ada +. (TIF) [file pone.0022667.s003.tif]

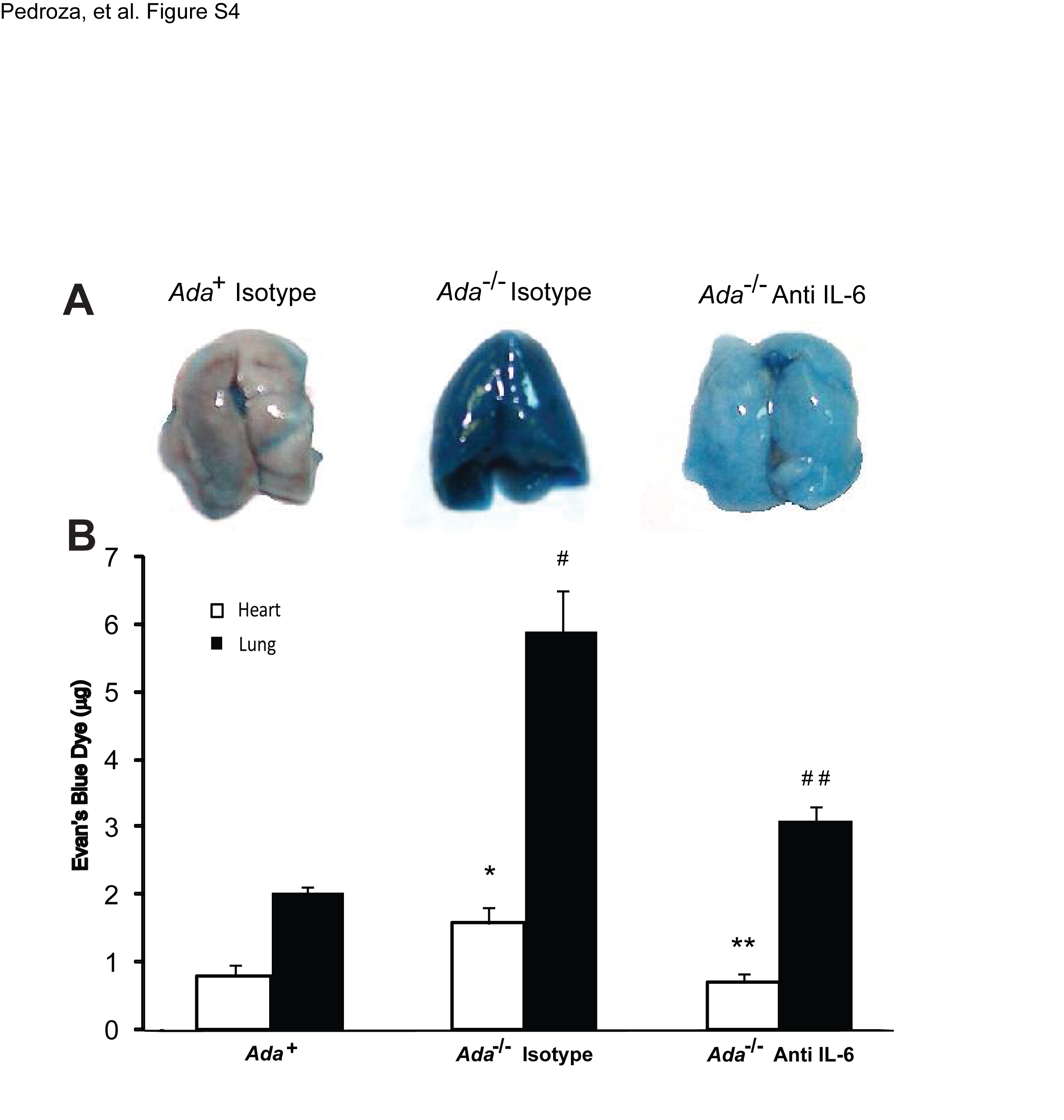

Supplement: Figure S4 — Vascular barrier function following treatment with IL-6 neutralizing antibodies. Ada+ and Ada -/- mice were treated subcutaneously with IL-6 neutralizing antibodies as described in the methods. On day 43, mice were injected with Evans blue dye. 4 hrs later, mice were anesthetized, perfused and lungs and hearts were removed. (A) Whole mounts demonstrating increased Evans blue tissue uptake in the lungs of Ada-/- mice and reduced uptake in Ada-/- treated with IL-6 neutralizing antibodies (Anti IL-6). Images are representative of 4 mice. (B) Organs were extracted in formamide and dye concentrations were determined in lungs and hearts. Data are presented as mean µg/lung ±SEM. n = 4. *, p≤0.05 Ada + vs Ada -/- and #, p≤0.05 Ada -/- vs Ada -/- + Anti-IL-6. (TIF) [file pone.0022667.s004.tif]

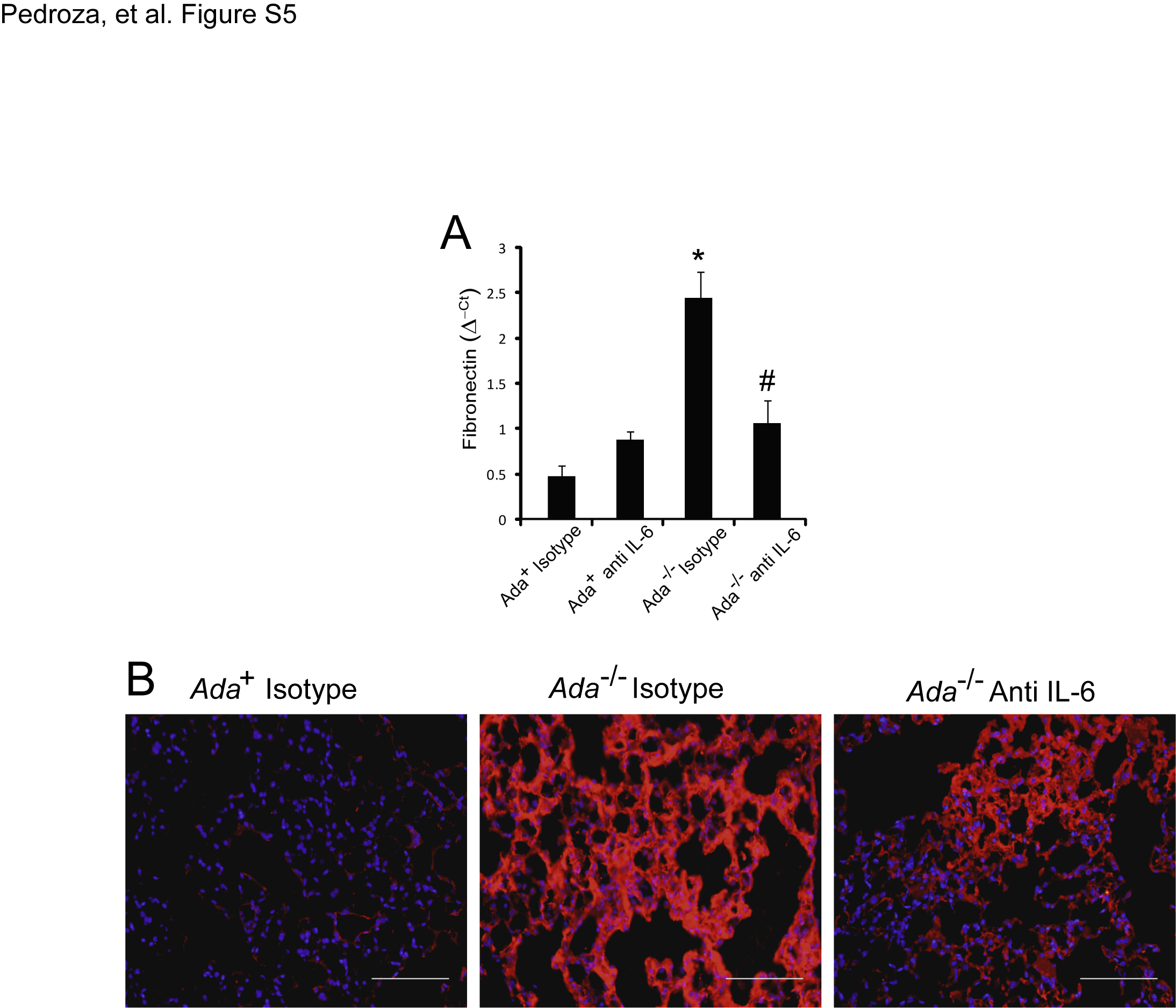

Supplement: Figure S5 — Decreased fibronectin in the lungs of Ada -/- mice treated with IL-6 neutralizing antibodies. Analyses were on postnatal day 43. (A) Whole-lung fibronectin transcript levels were measured using quantitative RT-PCR. Data are presented as mean normalized 18S rRNA transcript levels (Δ−ct) ±SEM, n≥4. *, p≤0.05 Ada + vs Ada -/- and #, p≤0.05 Ada -/- vs Ada -/- + Anti-IL-6. (B) Decreased fibronectin deposition visualized by fibronectin immunofluorescence (red) blue represents dapi stained nuclei. Images are representative of 8 animals from each group. Scale bars: 200 µm. (TIF) [file pone.0022667.s005.tif]

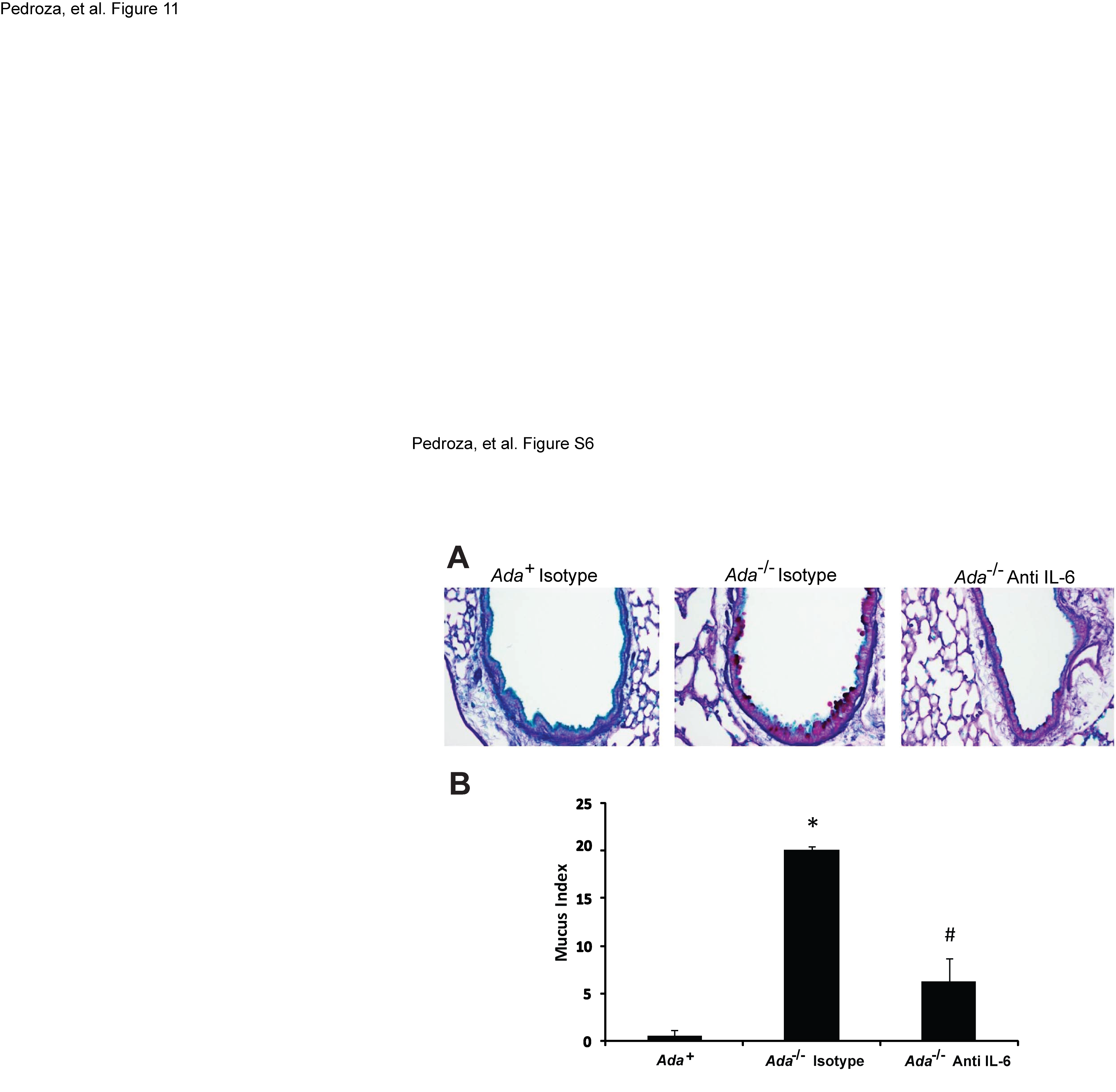

Supplement: Figure S6 — Decreased mucus cell metaplasia following treatment with IL-6 neutralizing antibodies. Ada+ and Ada -/- mice were treated subcutaneously with an IL-6 neutralizing antibodies as described in the methods. Lung sections from day 43 mice were subjected to periodic acid-Schiff (PAS) staining that stained mucus pink. (A) Representative views of bronchial airways from 6 mice in each group. (B) Image Pro software was used to quantify the degree of periodic acid-Schiff staining in bronchial epithelial cells and data are presented as a mean Mucus Index ±SEM. n = 6; p≤0.05 Ada + vs Ada -/- and #, p≤0.05 Ada -/- vs Ada -/- + Anti-IL-6. (TIF) [file pone.0022667.s006.tif]
